# What happens to conservatively managed chronic subdural haematoma

**DOI:** 10.1007/s00701-025-06577-6

**Published:** 2025-06-07

**Authors:** Jack Read, Ellie Edlmann

**Affiliations:** 1 University of Exeter Medical School, Exeter, UK; 2 Peninsula Medical School, Faculty of Health, University of Plymouth, Plymouth, UK; 3 Department of Neurosurgery, University Hospitals Plymouth NHS Trust, Plymouth, UK

**Keywords:** Chronic subdural, Aging, Elderly, Head injury

## Abstract

**Background:**

Chronic subdural haematoma (CSDH) is a common neurosurgical problem, increasingly prevalent due to an ageing population. Conservative management is an option for asymptomatic or mild cases, though data on outcomes is limited.

**Aim/Objective:**

This study aims to address critical gaps in understanding around conservatively managedCSDH and whether there are features that can predict progression and the longer-term prognosis of these patients.

**Methods:**

A retrospective cohort study was performed at a major trauma centre in the UK. All patients with chronic subdural haematoma referred from March 2019 to March 2021 were included and grouped into surgical or conservative management. Demographic, outcome, and radiological data were collected from patient records and compared.

**Results:**

Of 289 patients, 90 had surgery and 199 were managed conservatively. Conservative patients were older (84 vs 77 years, p < 0.0001), had more comorbidities (4 vs 2, p < 0.0001), higher frailty (CFS > 6: 45% vs 10%, p < 0.0001), and greater anti-thrombotic use (57% vs 42%, p = 0.0175). Mortality was significantly higher in the conservative group at all time points including one month (16% vs 2%, p < 0.0001), one year (42% vs 12%, p < 0.0001) and two years (55% vs 21%, P < 0.0001). Surgical patients had significantly greater midline shift (10 mm vs 2.6 mm, p < 0.0001), and haematoma depth (24 mm vs 11 mm, p < 0.0001). Only 3% of conservative patients crossed over to surgery.

**Conclusion:**

Our study highlights the high mortality rate in conservatively managed CSDH, with frailty as a key indicator for early death. The low crossover to surgery questions the necessity of radiological monitoring in conservatively managed CSDH.

## Introduction

Chronic subdural haematoma (CSDH), the accumulation of blood and fluid between the dura and arachnoid of the brain, presents a significant challenge in neurosurgery [12, 24]. Predominantly affecting the elderly, its incidence is increasing, driven by an aging population and the widespread use of anticoagulants [24, 29, 35, 43]. CSDH cases are projected to rise 50% by 2040, particularly amongst the oldest patients [29, 35].

Computed tomography (CT) is the primary diagnostic modality for CSDH, with characteristic hypodense crescent-shaped collections over the brain [25]. CSDH can also be isodense or hyperdense, due to newer bleeding [24, 25]. Radiographic diagnosis can be challenging when distinguishing between hypodense CSDH and subdural effusion or hygroma [14, 25]. There is likely to be a continuum between brain shrinkage resulting in enlarged CSF spaces over the brain and a defined collection reported as a hygroma or CSDH.

Management is typically based on CSDH size and the severity of presenting symptoms [19, 24, 26]. Patients usually present with confusion, headache, gait disturbance, hemiparesis and eventually ​reduced consciousness [24]​. Patients with sizeable symptomatic collections require surgical evacuation [24, 26]​. Surgery is not without risk and exposes patients, many of whom are frail, to complications such as infection, seizures, pneumocephalus and new neurological deficits[24, 40]. Though most recover well after surgery, 9–14% face recurrence [5, 9, 19]. Adjunctive therapies such as corticosteroids have been shown to reduce recurrence, although they are associated with increased adverse effects and poor outcome [21, 39, 44].

Patients who are asymptomatic, have mild symptoms, orare unfit for surgery, may have non-surgical, or conservative, management [15, 26, 39]. Standalone pharmacotherapy such as tranexamic acid (TXA), corticosteroids, angiotensin converting enzyme inhibitors (ACE-i), and atorvastatin have been trialled with limited or no evidence on their overall benefit [39, 41].

Increased utility of CT head scanning means that smaller and incidental CSDHs are more frequently diagnosed. Conservative management strategies (excluding pharmacotherapy), often termed ‘wait-and-watch’, is favoured in such patients, emphasising observation and symptomatic care [15, 41].Despite its frequency, there is limited published data on the outcomes for these patients. A systematic review by Foppen et al. in 2023, encompassing 11 studies with a total of 1019 patients reported a success rate of 66% for watch-and-wait therapy [15]. This review highlighted that while conservative management can be effective, the patient population remains diverse. This variability underscores the need for a deeper understanding of the factors influencing the outcomes in conservatively managed CSDH. Predictive factors remain contentious; while factors like haematoma volume [45, 47] and hypodensity on CT scan [20, 47] are implicated, studies lack consensus [15].

This study aims to address critical gaps in understanding around conservatively managedCSDH. Specifically, it seeks to compare baseline patient characteristics between conservatively and surgically managed CSDH, whether there are features that can predict progression and the longer-term prognosis of these patients.

## Methods

### Study design and participants

We conducted a single-centre, retrospective cohort study at a tertiary centre in the UK which has a catchment population of 1.4 million. The study included all patients referred with a neurosurgical diagnosis of CSDH between March 2019 and March 2021. Patients were divided into two groups: those who were admitted for surgical management and those who received conservative management. Conservative management was defined as a watch-and-wait approach without any adjunctive therapy and no routine follow-up. Surgery is performed under general anaesthetic as standard in our region and middle meningeal artery embolization was not utilised at the time of the study. The treatment decision was made by the accepting consultant on the basis of referral information and imaging, only patients in the regional hospital may have been directly reviewed by the registrar.

### Variable baseline data

Baseline information was collected from an electronic regional referral database, which elicits clinical information from the local referring team and is the only method of referring. Inclusion as a CSDH was determined by the neurosurgical registrar receiving the referral and categorising it as this. Those categorised as hygromas by the receiving neurosurgeon were excluded. Baseline data included demographics, co-morbidities, Rockwood Clinical Frailty Scale (CFS) score, anti-thrombotic use, mechanism of injury, symptoms and Glasgow Coma Scale (GCS). The Charlson Comorbidity Index (CCI) is an assessment tool used to predict 10-year mortality in patients with multiple co-morbidities, and was calculated from the history [8]. Survival data up to 2 years was checked.

Rockwood clinical frailty scale scores are commonly used in the UK to quantify frailty, evaluating comorbidity, function, and cognition ranging from 1 (very fit) to 9 (terminally ill). As per the literature, we used a CFS of 6 and above to describe patients as having a higher level of frailty [10, 37].

### Radiological data

Radiological imaging for all patients was analysed. Data included; haematoma laterality (unilateral versus bilateral), side (left/right), density on CT, maximum depth, and degree of midline shift. Haematoma depth was measured on multiple slices and the largest value taken. Midline shift was measured from the falx cerebri to the septum pellucidum [2]. Density of the haematoma was classified into hypodense, isodense, hyperdense, and mixed density if there was a combination of density types**.** All radiological reports were reviewed and noted if they reported hygroma rather than CSDH (despite neurosurgeon’s diagnosis). Imaging data was linked across the catchment region and all patients were searched for any follow-up imaging available in the study period.

### Statistical analysis

All data was stored anonymously. Descriptive statistics were calculated and data was compared using an unpaired t test if parametric and Mann–Whitney if nonparametric. Categorical data were tested using the chi-square test. Mortality rates were calculated and adjusted for age using direct age standardisation, and compared using a Mantel–Haenszel test. Survival data were adjusted for age and plotted using a Cox proportional hazard regression curve. Statistical tests were conducted using GraphPad Prism (v10) and R (v4.5.0). A p value of less than 0.05 was considered significant. There were no missing outcome data points or patients lost to follow up; however three patients referred during the study period were excluded due to absence of NHS number.

### Ethics statement

This study was registered and approved by the institutional audit department. As the study involved anonymised data collected as part of routine care, patient consent was not applicable.

## Results

A total of 289 patients were referred during the study period. 90 patients were accepted for surgery, and 199 patients were managed conservatively. Six conservative patients (3%) subsequently had surgery.

### Baseline characteristics

Baseline characteristics are outlined in Table 1. The mean age of all patients was 82 years. Conservative patients were significantly older than surgical patients (mean 84 versus 77 years, *p* < 0.0001). 192 (66%) patients were male and the most common mechanism of injury was a fall from less than 2 m (82%).

**Table 1:** Baseline patient characteristics

Characteristic	Conservative (*n* = 199)	Surgical (*n* = 90)	Total (*n* = 289)	*P* value
Mean Age (SD)	**84 (10.2)**	**77 (12.0)**	**82 (11.2)**	** < 0.0001**
Male (%)	135 (68)	57 (63)	192 (66)	0.4526
Mechanism, n (%)				
Fall < 2 m	160 (80)	76 (84)	236 (82)	
Blow	5 (3)	1 (1)	6 (2)	
Vehical incident	2 (1)	2 (2)	4 (1)	
Fall > 2 m	1 (0.5)	0	1 (0.4)	
Previous Head injury	0	1 (1)	1 (0.4)	
No History of Trauma	31 (16)	10 (11)	41 (14)	
Anti-thrombotics, n (%)				
All anti-thrombotics	**114 (57)**	**38 (42)**	**152 (53)**	**0.018**
Anti-platelet	42 (21)	19 (21)	61 (21)	0.999
Oral Anti-coagulant	**82 (41)**	**21 (23)**	**103 (36)**	**0.0033**
Frailty, n				
Median Frailty (IQR)	**5 (3–6)**	**3 (2–4)**	**4 (2–6)**	** < 0.0001**
Higher frailty, CFS > 5 (%)	**89 (45)**	**9 (10)**	**98 (34)**	** < 0.0001**
Lower frailty, CFS < 6 (%)	**110 (55)**	**81 (90)**	**135 (48)**	** < 0.0001**
Co-morbidities, n				
Median no. (IQR)	**4 (2–5)**	**2 (1–4)**	**3 (2–5)**	** < 0.0001**
Median CCI (IQR)	**5 (4–7)**	**4(3–6)**	**5 (4–7)**	** < 0.0001**
Neurological (%)	**92 (46)**	**25 (28)**	**117 (40)**	**0.0031**
Cardiac (%)	135 (68)	51 (57)	186 (64)	0.066
Vascular (%)	11 (6)	6 (7)	17 (6)	0.703
Endocrine (%)	49 (25)	15 (17)	64 (22)	0.131
Neoplastic (%)	39 (20)	13 (14)	52 (18)	0.291
Respiratory (%)	39 (20)	16 (18)	55 (19)	0.715
Gastrointestinal (%)	38 (19)	15 (17)	53 (18)	0.621
Genitourinary/Renal (%)	**50 (25)**	**7 (8)**	**57 (20)**	**0.0006**
Musculoskeletal (%)	**62 (31)**	**17 (19)**	**79 (27)**	**0.0303**
Symptoms, n (%)				
Gait disturbance	102 (51)	57 (63)	159 (55)	0.056
Confusion	66 (33)	32 (33)	98 (33)	0.930
Headache	**24 (12)**	**27 (30)**	**51 (18)**	**0.0002**
Hemiparesis	**13 (7)**	**24 (27)**	**37 (13)**	** < 0.0001**
Speech disturbance	21 (11)	11 (12)	32 (11)	0.675
Nausea/Vomiting	7 (4)	7 (8)	14 (5)	0.118
Asymptomatic	**37 (19)**	**4 (4)**	**41 (14)**	**0.0014**
Other	23 (12)	16 (18)	39 (14)	0.159
GCS				
Median GCS (IQR)	15 (14–15)	15 (14–15)	15 (14–15)	0.447
GCS 15 (%)	132 (66)	51 (57)	183 (63)	0.114
GCS 13 to 14 (%)	61 (31)	32 (36)	93 (32)	0.409
GCS 9 to 12 (%)	6 (3)	6 (7)	12 (4)	0.150
GCS < 8 (%)	0 (0)	1 (1)	1 (0.4)	0.136

Overall, 152 patients (53%) were taking an anti-thrombotic medication, with a significantly higher proportion in the conservative group compared to the surgical group (57% vs 42% *p* = 0.0175).

The median frailty of all patients was 4. Conservative patients had higher frailty than surgical groups (median CSF 5 vs 3, *p* < 0.0001)**.** Additionally, more conservative patients had a high level of frailty (CFS > 5) than surgical patients (45%, vs 10%, *p* < 0.0001).

Conservative patients had more comorbidities (median 4 vs 2, *p* < 0.0001) and a higher CCI (median 5 vs 4, *p* < 0.0001) than surgical patients. The critical co-morbidities in conservative patients were neurological (46% vs 28%, *p* = 0.0031), musculoskeletal (31% vs 19%, *p* = 0.0303), and renal/genitourinary (25% vs 8%, *p* = 0.0006). For neurological comorbidities, this was primarily driven by the rate of dementia (*n* = 40 vs *n* = 6), cerebrovascular disease (*n* = 33 vs *n* = 8), and Parkinson’s disease (*n* = 11 vs *n* = 2). Renal/genitourinary group comprised chronic kidney disease (*n* = 30 vs *n* = 5), benign prostatic hyperplasia (*n* = 8 vs *n* = 1), and urinary tract infections (*n* = 5 vs *n* = 0). Musculoskeletal comorbidities were primarily fractures (*n* = 14 vs *n* = 2), osteoarthritis (*n* = 11 vs *n* = 5), and joint replacements (*n* = 10 vs *n* = 2).

Both cohorts had a median GCS of 15. Headache (30% vs 12%, *p* = 0.0002), and hemiparesis (27% vs 7%, *p* < 0.0001) were more common in the surgical group, while asymptomatic cases were higher in the conservative group (19% vs 4%, *p* = 0.0014). Most symptomatic patients had more than one symptom (58%).

#### Mortality

**Fig. 1:**
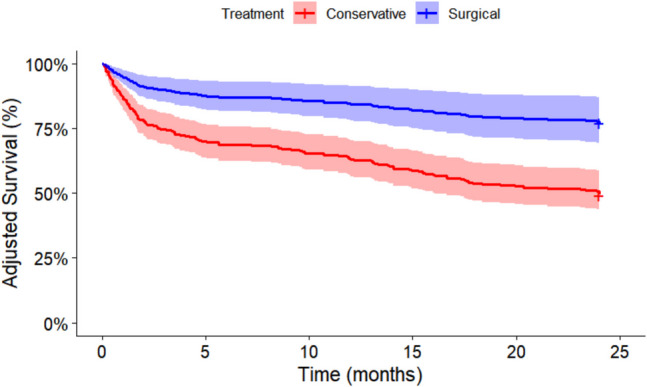
Age adjusted survival in conservative and surgically managed CSDH patients, up to 24 months

**Fig. 2:**
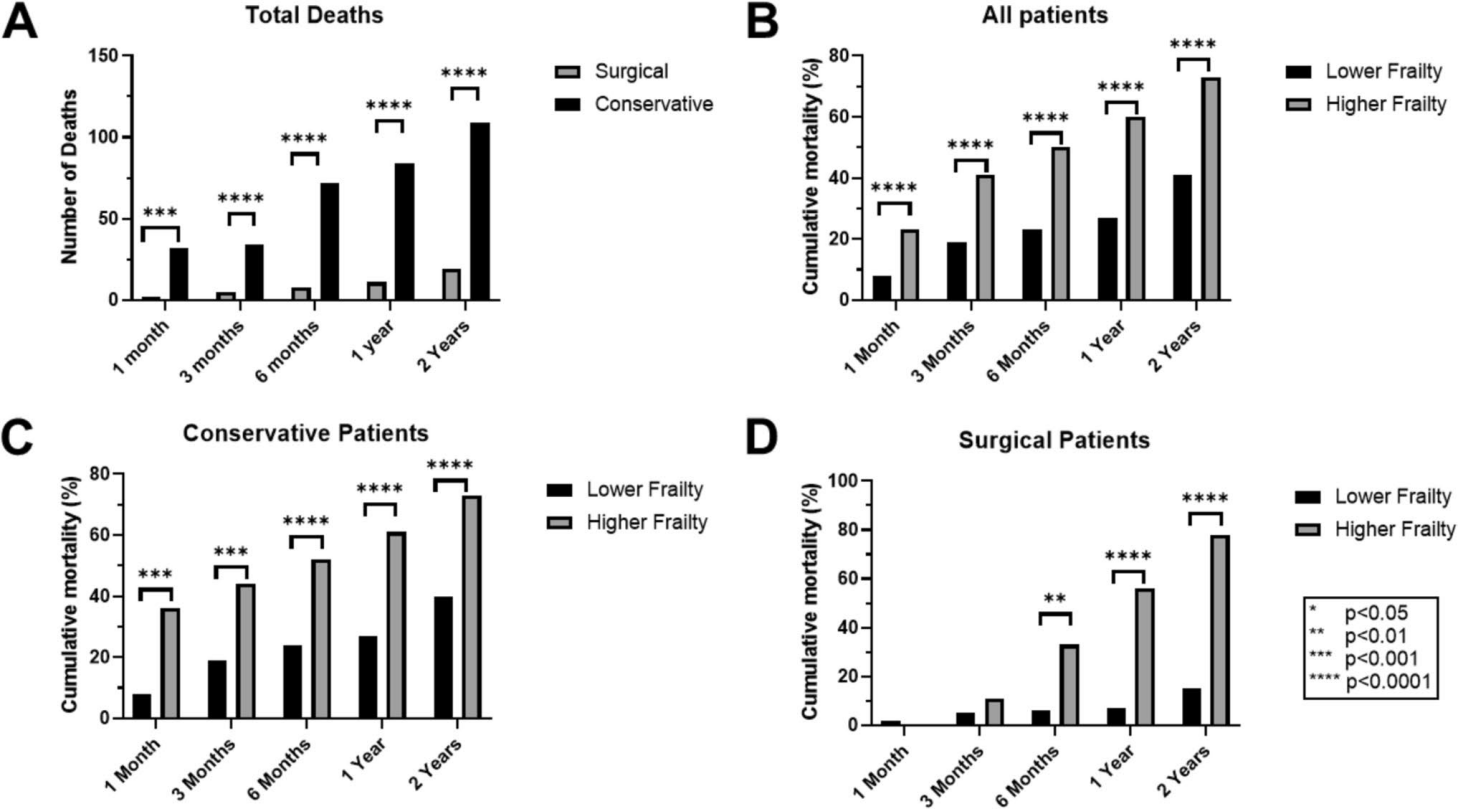
Frailty and Mortality-**A** Cumulative number of deaths across time periods in each group. **B** Cumulative deaths as a proportion of frailty group in all patients. **C** Cumulative deaths as a proportion of frailty group in conservative patients. **D** Cumulative deaths as a proportion of frailty group in surgical patients

The age-adjusted mortality rate was significantly higher in conservative patients compared to surgical patients at all time points (1, 3, 6 months, 1 and 2 years; see Fig. 1 and Table 2). Frailty significantly increased mortality in both groups, though impact on surgical patients was delayed until 6 months (Fig. 2).

**Table 2:** Mortality data from one month to two years in conservative and surgically managed CSDH. *AMR* = Age adjusted mortality rate

Time point	Conservative AMR	Surgical AMR	OR (95% CI)	*p*-value
1 Month	15%	3%	6.3 (1.5–26.2)	*p* = 0.0006
3 Months	29%	6%	6.5 (2.4–17.6)	*p* < 0.0001
6 Months	35%	10%	4.8 (2.2–10.8)	*p* < 0.0001
1 Year	40%	14%	4.3 (2.1–8.8)	*p* < 0.0001
2 Years	53%	25%	3.7 (2.0–6.7)	*p* < 0.0001

### Imaging characteristics

**Fig. 3:**
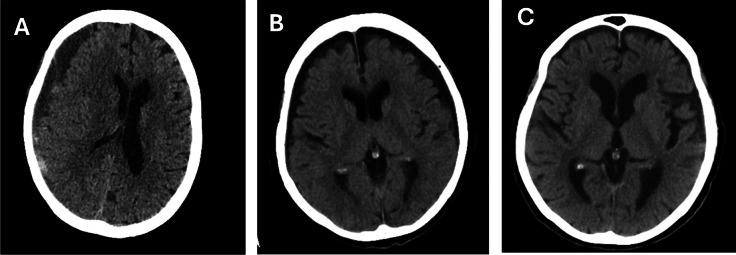
**A** 77-year-old patient managed conservatively (NOAC stopped) and remained asymptomatic until their death 3 months later from an unrelated cancer. Categorised as CSDH with mixed density. **B** 81-year-old patient with a fall managed conservatively, categorised as bilateral hygroma. **C** Same patient as in (b) with hygromas noted to have resolved on a repeat scan after 3 months

Imaging characteristics are detailed in Table 3. Significantly more conservative patients had radiologically-reported hygromas than surgical patients (*n* = 50, 25% vs *n* = 0, 0%, *p* < 0.0001). Examples of the difference between radiologically reported CSDH and hygromas are shown in Fig. 3.

**Table 3:** Imaging features (MLS = midline shift)

Imaging characteristics	Conservative (*n* = 199)	Accepted (*n* = 90)	Total (*n* = 289)	*P*-Values
Bilateral (%)	81 (41)	29 (32)	110 (38)	0.169
Hygroma reported (%)	**50 (25)**	**0 (0)**	**50 (17)**	** < 0.0001**
Left sided (%)	56 (28)	29 (32)	85 (29)	
Follow up scans (%)	**119 (60)**	**78 (87)**	**197 (68)**	** < 0.0001**
Follow up within 1 week (%)	**38 (32)**	**54 (70)**	**92 (47)**	** < 0.0001**
Median days to follow up scan (IQR)	**15 (4–54)**	**4 (3–8)**	**7 (3–32)**	** < 0.0001**
Mean measures (SD)				
MLS, mm	**2.6 (3.5)**	**10.0 (4.0)**	**5.1 (5.1)**	** < 0.0001**
Unilateral depth, mm	**10.8 (6.3)**	**24.0 (7.5)**	**15.3 (9.2)**	** < 0.0001**
Bilateral depth, mm	**12.7 (5.6)**	**23.1 (7.7)**	**15.4 (7.7)**	** < 0.0001**
Right-sided depth	11.0 (5.4)	20.0 (8.8)	13.4 (7.6)	
Left-sided depth	10.7 (4.8)	16.9 (7.2)	12.4 (6.2)	
Density on CT, n (%)				
Hypodense (%)	53 (26)	24 (27)	76 (26)	0.065
Isodense (%)	20 (10)	2 (2)	22 (8)	
Mixed density (%)	121 (61)	61 (68)	182 (63)	
MRI (%)	3 (2)	5 (6)	8 (3)	
CSDH vs Hygroma	**CSDH (n = 239)**	**Hygroma (n = 50)**	**Total (n = 289)**	**p-value**
Bilateral (%)	**81 (34)**	**29 (58)**	**110 (38)**	**0.0014**
Left-sided (%)	74 (31)	11 (22)	85 (29)	
Mean measures (SD)				
MLS, mm	**5.7 (5.1)**	**0.9 (1.9)**	**5.1 (5.1)**	** < 0.0001**
Unilateral depth, mm	**16.2 (9.3)**	**8 (2.8)**	**15.3 (9.2)**	** < 0.0001**
Bilateral depth, mm	**17.0 (8.2)**	**11.1 (3.3)**	**15.4 (7.7)**	**0.0003**
Right-sided depth	14.7 (8.2)	9.8 (3.5)	13.4 (7.6)	
Left-sided depth	13.4 (6.7)	9.6 (3.2)	12.4 (6.2)

Midline shift was greater in the surgical group than in the conservative group (10 mm versus 2.6 mm, p < 0.0001). Maximum depth was greater in the surgical group than in the conservative group for both unilateral (24 mm vs 11 mm, *p* < 0.0001), and bilateral haematomas (23.1 mm vs 12.7 mm). Haematoma density on CT scan showed no significant difference, with mixed density being the most common (*n* = 182, 63%). Eight patients (3%) had MRI instead of CT scans.

Significantly more hygromas were bilateral than CSDH (*n* = 29, 58% vs *n* = 81, 34%, *p* = 0.0014). Maximum midline shift was greater in CSDH than hygromas (5.7 mm vs 0.9 mm, *p* < 0.0001). CSDH has a greater maximum depth than hygromas for unilateral cases (16.2 mm vs 8.0 mm, *p* < 0.0001) and bilateral cases (17.0 mm vs 11.1 mm, *p* = 0.0003).

Follow up scans were available in 68% of patients, follow up imaging was unavailable for one patient due to being from out of area. Significantly more surgical patients had follow up scans within a week of referral (70% vs 32%, *p* < 0.0001) and had a shorter median time to follow up scan (4 d vs 15 d, *p* < 0.0001).

## Discussion

This retrospective study on a single UK tertiary centre highlights differences in baseline characteristics, imaging features and mortality between conservatively and surgically managed CSDH patients. We had an annual crude operative rate of 3.2 per 100,000 and non-operative rate of 7.1 per 100,000 in our region, this is similar to that reported in previous studies [43].

We identified only nine studies examining conservatively managed CSDH patients in the literature, including two with fewer than 50 patients and one systematic review, suggesting a lack of evidence in this field [15].

In our data, the conservative cohort was significantly older, frailer, and carried a greater burden of comorbidities at presentation, compared to the surgical cohort. Specifically, these patients exhibited elevated CFS and CCI scores, indicating a greater health burden. The greatest difference was seen in the burden of neurological disease (dementia and stroke), chronic kidney disease and musculoskeletal disease (fractures and osteoarthritis). Collectively, these comorbidities exacerbate frailty in patients, negatively impacting physical and cognitive function, mobility, and independence [6, 30, 36, 38]. Cardiovascular disease was the most common co-morbidity across all patients (64%), reflected with high rates of anti-thrombotic use (53%). The rate of OAC use was significantly higher in the conservative cohort, which may reflect the increased stroke history identified. The likelihood of AF and other stroke risk factors are also likely to be increased in the conservative cohort as it is significantly older, with current evidence supporting anti-coagulation for such patients [46]. Overall, our findings highlight high levels of frailty and co-morbidity across the CSDH population, underscoring the need for early geriatric input and a multidisciplinary approach, as has previously been proposed [42].

Gait disturbance and confusion were common across both cohorts, and highlights the non-specific nature of these symptoms. Hemiparesis and headache were more common in the surgical cohort, reflecting symptoms of brain compression indicating surgery. Nearly 1/5 th of the conservatively managed patients were asymptomatic, supporting the principle that asymptomatic or mild CSDH are suitable for conservative management [15]. This is reflected in the imaging analysis, with greater midline shift and haematoma depth in surgical patients, as one would expect. Smaller CSDHs in the conservative cohort are much less likely to be causing symptoms, with other co-existing pathology the true cause of symptoms. In many conservative cases (25%), hygromas were reported, despite the neurosurgeon categorising them as CSDHs, highlighting the difficulty in distinguishing these pathologies. Hygromas are considered to be of CSF density, but hypodense CSDHs can appear similar (Fig. 3). The significant difference in size between hygromas and CSDH may indicate that small, hypodense collections are more likely to be hygromas than CSDHs. It is also theorised that hygroma is a stage during the formation of CSDH, and both are linked by the underlying risk factor of brain atrophy, with shrinkage allowing a potential space for fluid or blood to accumulate [27]. Whilst increasing the risk of subdural haemorrhage, brain atrophy is also correlated with cognitive decline and increased frailty [3, 18, 23]. We think this study shows that the diagnosis of a CSDH for conservative management or even hygroma, should be a red flag signifying underlying brain atrophy and contributing to frailty.

The overall mortality of CSDH patients, surgical and conservative, surpasses that of the general population. In England, the predicted life expectancy for a male aged 84 is 6 years, whereas in the conservative cohort, with the same mean age, over 50% had died within 1 year [31]. This is worse than other similar conditions often related to frailty, such as fractured neck of femur, which is considered to have a 20–35% 1-year mortality rate [17]. Even studies on frail patients attending the emergency department report 30-day mortality rates of 5–10% compared to 16% in the conservative CSDH cohort [22]. Frailty significantly impacted mortality, especially in conservative patients, aligning with previous studies identifying frailty as a key prognostic factor in CSDH [4, 28, 32]. In surgical patients, frailty was not significantly associated with mortality early on, suggesting surgery itself is not resulting in mortality in these patients. However, by six months, frailty-related mortality increased in this group, reinforcing that frailty is a critical determinant of outcomes in the longer term.

Only 1% of the conservative cohort required subsequent surgery, a rate similar to the 0.9% reported by Parry et al. [33]. This low crossover rate suggests that routine radiological surveillance is not necessary for most patients if their clinical status remains stable, supporting a shift toward symptom-guided follow-up for conservatively managed patients [33]. It is perhaps surprising that more CSDHs did not progress, given the understanding in pathophysiology of CSDH as a continued cycle of inflammation [11]. Despite this, clearly not all CSDHs are progressive and most small CSDHs remain static or resolve over time. It may be that the inflammatory response is more self-limiting in this early phase, but more research is needed.

When comparing to previous studies on the conservative management of CSDH, our cohort was slightly older, with a mean age of 82 compared mean ages ranging from 65–81 years [7, 33, 34]. This difference may reflect the broader trend of an ageing CSDH population. GCS scores were similar to prior studies, suggesting comparable initial injury severity across cohorts [15, 16, 20, 45]. However, we found that CFS, a significant prognostic factor in our cohort, has not been consistently reported in previous literature. This lack of frailty data limits comparisons across studies, but underscores our finding that frailty plays a crucial role in management strategy for CSDH. Similarly, our cohorts median CCI score of 5 aligns with the mean CCI of 5 reported by Parry et al.[33]. Importantly, our study is the only one to compare both CCI and CFS between treatment groups, showing significantly higher scores in conservatively managed patients, highlighting why their mortality is significantly higher than for surgical patients.

Our imaging findings align with the broader literature on CSDH, though differences in study design affect comparability. While MLS was not deemed significant in studies by Foppen [16], Zhang [47], and Wang [45], our study found significantly greater MLS in the surgical cases. This may be due to the patient selection in these studies. Zhang included only mild CSDH cases and excluded 73% of these patients for reasons such as statin or anticoagulant use, incomplete records, and follow-up – yielding a narrow cohort [47]. Similarly, Wang’s study excluded patients on antithrombotic or statin medications, which are common in CSDH patients, limiting generalisability [45]. In contrast, our study included all CSDH patients, therefore providing a more realistic representation of the range of CSDH patients. Our findings on haematoma depth being significantly greater in surgical patients is unsurprising and supported by the literature, with volume likely an even more sensitive measure than depth [15, 16, 45, 47]. This implies that conservative patients are generally non-surgical due to size, rather than large collections in patients managed palliatively.

Our study’s 30-day mortality rate of 12% (16% for conservative patients, 2% for surgical patients) is notably higher than the 5% reported by Foppen et al. [16] (8% conservative, 2% surgical cross-over). However, direct comparison is challenging due to differences in patient selection. Foppen’s study excluded patients with withdrawn therapy, likely those with poor prognoses or deemed too frail to survive, essentially removing the most vulnerable patients from their sample. In contrast, our cohort included all referred CSDH patients, giving a broader representation of CSDH cases and reflecting the frailty and diversity of CSDH patients. With 72% of those who died within 30 days classified as highly frail, our findings further highlighting the impact of frailty on outcomes among conservatively managed patients.

Reported crossover rates from conservative management to surgery range from 1 to 93% [1, 7, 15, 16, 20, 33, 34, 45, 47]. In our study, only 3% required subsequent surgery. Differences likely reflect studies with higher crossover rates likely involve monitoring patients who, at our centre, would typically be managed surgically in the first instance. Our standard practice would be to offer surgery to symptomatic CSDH with evidence of mass effect on imaging, unless there was a strong reason not to operate. Ultimately, the primary reason for cross-over from conservative management to surgery, as stated in the literature, is deteriorating clinical status, accounting for up to 97% of cases [16]. This further emphasises that surveillance imaging is unnecessary for asymptomatic patients.

It is important to consider both the threshold and philosophy around CSDH treatment when reflecting on our data. The high rate of conservatively managed CSDH partly reflects a growing population of older people with brain atrophy where the subdural collections may just be hygromas and therefore would never be considered surgical. None-the-less many sizeable CSDHs resolved without intervention. This is particularly relevant in the age of MMAE, where some trials have included patients with small and/or minimally symptomatic collections, the majority of which could have resolved without any treatment [13]. Patient selection for MMAE is critical, and it is essential that more data is reported on non-operative CSDH such that we can understand the natural history further and be certain that interventions are only performed where necessary. Whilst the mortality rate is high in the conservative arm, few patients are refused surgery on the basis of co-morbidity, as most are fit for a short operation with an excellent recovery rate. Thus, we feel the excess mortality reported in this study largely relates to their underlying global health and that CSDH is a marker of frailty rather than the cause of it. Therefore, interventions in this cohort (including MMAE) would be unlikely to change the outcome, and rather, better holistic care to optimise quality of life is important. Further data in this field, and stratification of treatment interventions by frailty may be helpful in future trials.

### Limitations of this study

This study is limited by its retrospective, single-centre design which may introduce selection bias and limit generalisability due to variations in clinical practice. There are no standard guidelines on when a CSDH should be accepted for surgery, therefore this is at the surgeon’s discretion. There will be selection bias of not operating on highly co-morbid and frail patients, contributing to their higher mortality. However, the threshold for operating is relatively low, and most of the conservative cohort had smaller CSDH’s with no/minimal shift, therefore judged not to require surgery rather than to be palliative.

Whilst we were able to access follow-up imaging and clinical data from across our region, it is possible that any patient visiting from out-of-region was lost-to-follow-up. In our experience, in the multi-morbid age group, few patients are travelling from out-of-region and therefore we anticipate very few patients were lost-to-follow-up. Finally, our study lacks detailed morbidity and functional data, limiting insights into long-term outcomes and quality of life. This highlights the need for a prospective, multi-centre study to explore these differences further and validate our findings.

## Conclusion

Our study highlights the importance of frailty in understanding prognosis and outcomes in conservatively managed CSDH. This should drive early geriatric input to help address the complex health needs of these patients and improve information giving for patients and their families. Low crossover rates suggest symptom guided follow-up is sufficient for stable patients, reducing the need for routine imaging. Further research is needed to validate these findings and explore functional outcomes in greater detail, with the aim to refine care strategies for conservatively managed patients with CSDH.

## Data Availability

No datasets were generated or analysed during the current study.
